# A Novel, Cleaved Probe-Based Reverse Transcription Loop-Mediated Isothermal Amplification Method for Specific and Sensitive Detection of Porcine Deltacoronavirus

**DOI:** 10.3389/fvets.2022.896416

**Published:** 2022-06-23

**Authors:** Haiyan Shen, Songqi Wang, Jun Huang, Qijie Lin, Chunhong Zhang, Zhicheng Liu, Jianfeng Zhang, Ming Liao

**Affiliations:** ^1^Maoming Branch Center of Guangdong Laboratory for LingNan Modern Agricultural Science and Technology; Key Laboratory of Livestock Disease Prevention of Guangdong Province, Scientific Observation and Experiment Station of Veterinary Drugs and Diagnostic Techniques of Guangdong Province, Ministry of Agriculture and Rural Affairs; Institute of Animal Health, Guangdong Academy of Agricultural Sciences, Guangzhou, China; ^2^National and Regional Joint Engineering Laboratory for Medicament of Zoonoses Prevention and Control; Key Laboratory of Zoonoses, Ministry of Agriculture; Key Laboratory of Zoonoses Prevention and Control of Guangdong Province; Key Laboratory of Animal Vaccine Development, Ministry of Agriculture; College of Veterinary Medicine, South China Agricultural University, Guangzhou, China; ^3^College of Life Science and Engineering, Foshan University, Foshan, China

**Keywords:** porcine deltacoronavirus, CP-RT-LAMP, ribonuclease H2, specific and sensitive detection, point-of care testing

## Abstract

Porcine deltacoronavirus (PDCoV) causes watery diarrhea, vomiting, and 30–40% mortality in newborn piglets. A simple, rapid, and sensitive method for PDCoV detection is valuable in its surveillance and control. Here, we developed a novel, cleaved probe-based reverse transcription loop-mediated isothermal amplification (CP-RT-LAMP) method for PDCoV detection. A cleaved probe with a ribonucleotide insertion that targeted the N gene of PDCoV was designed. During the reaction, the enzyme ribonuclease H2 is activated only when the cleaved probe is perfectly complementary to the template, leading to the hydrolytic release of a quencher moiety and signal output. This method can be easily used on a real-time fluorescence quantitative equipment or an on-site isothermal instrument combined with a smartphone. The specificity assay showed no cross-reactivity with other porcine enteric pathogens. This method had a detection limit of 25 copies/μL, suggesting comparable sensitivity with reverse transcription quantitative PCR (RT-qPCR). In detecting 100 clinical samples (48 fecal swab specimens and 52 intestinal specimens), the detection rate of the CP-RT-LAMP method (26%) was higher than that of RT-qPCR (17%). Thus, it is a highly specific and sensitive diagnostic method for PDCoV, with a great application potential for monitoring PDCoV in the laboratory or point-of-care testing in the field.

## Introduction

Porcine deltacoronavirus (PDCoV) is an enveloped, single-stranded, positive-sense RNA virus that belongs to the genus Deltacoronavirus in the family Coronaviridae (1). PDCoV was first identified in Hong Kong, China, in 2012 and subsequently detected on swine farms in the United States, Canada, South Korea, China, Thailand, Laos, and Vietnam (2). It causes an acute, highly contagious, and devastating enteric disease characterized by diarrhea and vomiting, dehydration, and a high number of deaths in neonatal piglets (3–5). PDCoV infection has become prevalent in pig farms around the world, causing enormous economic losses in multiple countries, and remains a serious challenge to the swine industry. Moreover, the broad receptor engagement of PDCoV may potentiate its diverse cross-species transmissibility (6). Recent studies have reported that calves and chickens are also susceptible to PDCoV (7, 8). Furthermore, there have been independent cases of porcine deltacoronavirus infection among Haitian children in 2021 (9). Therefore, it is of great significance to improve its detection and surveillance.

Clinically, PDCoV infection and resultant intestinal diseases may lead to clinical symptoms similar to those observed in porcine epidemic diarrhea virus (PEDV) and transmissible gastroenteritis virus (TGEV) infections, including diarrhea, dehydration, and excessive vomiting, with high mortality especially in neonatal piglets (1). These clinical diseases and lesions are indistinguishable, and specific laboratory diagnostic testing is imperative to differentiate PDCoV infection and other intestinal diseases in pigs. Therefore, a specific and sensitive diagnosis of the disease is essential for its prevention and control. To date, various diagnostic methods have been developed to diagnose PDCoV infection in pigs. These methods include virus neutralization test, indirect fluorescent antibody assay, and enzyme linked immunosorbent assay (10–12). However, they require specialized and expensive equipment or long reaction times. Although reverse transcription PCR (RT-PCR) and real-time PCR are suitable for rapid detection of PDCoV (13, 14), they involve a complex detection process and require trained technicians, which is not suitable for on-site applications.

Loop-mediated isothermal amplification (LAMP) amplifies nucleic acids under isothermal conditions and provides a potentially effective tool for rapid and on-site detection of viruses. This novel gene detection technique requires 40–60 min to detect PDCoV at 60–65°C, which makes it cost effective and time saving (15). It can be performed under constant temperature conditions without the need for advanced instruments and professional operators. However, detection of products based on turbidity or fluorescent dyes in LAMP is associated with the detection of non-specific amplification products (16).

In the present study, we developed a novel, one-step, closed-tube LAMP method for the specific detection of PDCoV, termed as cleaved probe-based reverse transcription loop-mediated isothermal amplification (CP-RT-LAMP). A cleaved probe with a ribonucleotide insertion that targeted the N gene of the PDCoV was designed on the basis of the original primer sets. The enzyme ribonuclease H2 (RNase H2) is only activated when the probe is perfectly complementary, leading to the hydrolytic release of a quencher moiety and thus an amplified signal (17). In addition, CP-RT-LAMP can not only monitor in real time but prevents false positive findings. Therefore, it is a rapid, sensitive, and specific tool for PDCoV detection, which is of great significance for the timely diagnosis, prevention, and control of PDCoV infection.

## Materials and Methods

### Primers and Probes

To ensure good detection ability of primers used in the CP-RT-LAMP method, the complete genome sequence of PDCoV was retrieved from GenBank and analyzed, and the conserved region of PDCoV N gene was selected to design primers. Five primers and a cleaved probe were designed using Primer Explorer V5. The primers comprised two outer primers F3 and B3, two inner primers FIP and BIP, and a loop F primer. Primers and the cleaved probe were synthesized by Sangon Biotech Co. Ltd (Shanghai, China). The detailed information of the primers and probes used in the CP-RT-LAMP is provided in Table 1.

**Table 1:** Primer and probe sequences of the cleaved probe-based reverse transcription loop-mediated isothermal amplification (CP-RT-LAMP) assay.

**Name**	**Sequence (5′ → 3′)**
F3	CCAGGAAACGCGACCAATC
B3	TGGCCAGCGAAAAGCATT
FIP	CGAGACCGGTTGCCAAATACCTCGTAAGACCCAGCATCAAGC
BIP	GCCAATGTCGGCTCTGCAGACAGGCACATGTCTGGCTAG
LoopF	GGGTAAAGTCCGCTTGGGA
probe	FAM-TGAGAAGACGGGTATGG***C*** (RNA) TGATC-BHQ1

### Virus Strains and Field Samples

PDCoV, swine acute diarrhea syndrome coronavirus (SADS-CoV), porcine reproductive respiratory syndrome virus (PRRSV) strain JXA1, pseudorabies virus (PRV) strain HB-98, classical swine fever virus (CSFV), porcine circovirus type 2 (PCV2), swine transmissible gastroenteritis virus (TGEV), and porcine epidemic diarrhea virus (PEDV) were preserved in our laboratory. All samples, including feces and intestinal contents, were collected from suckling piglets born to production sows and piglets from pig farms with acute diarrhea outbreaks in South China during 2019–2021.

### Construction of Standard Recombinant Plasmids

Total RNA was extracted using DNA/RNA Extraction Kits (TianLong Science and Technology Co., Ltd., China) according to the manufacturer's instructions. The amplified N gene of PDCoV was cloned into the pGEM-T Easy vector (Promega, Cat. A3600, A3610), which was used for the standard plasmid preparation. Plasmid DNA was extracted using the Plasmid Mini Kit I (Omega Bio-tek, Norcross, GA, USA) following the manufacturer's instructions. The purified recombinant plasmid pGEM-T-N was quantified by spectrophotometric analysis.

### Preparation of RNA Standard

The PCR product of the standard recombinant plasmid pGEM-T-N containing the RNA polymerase promoter region and the full-length N gene of PDCoV was used as template for *in vitro* transcription using the mMESSAGE mMACHINE® Kit (Life Technology, AM1344). Then, lithium chloride precipitation was used to remove unbound nucleotides and most of the protein, then RNA was recovered and purified. The RNA standard copy number was calculated, and RNA samples were stored at −80°C until further use.

### Establishment and Optimization of the Basic Reaction System

The CP-RT-LAMP basic reaction system was established as described previously (18) and effectively improved as follows: the system included 2.5 μL of buffer (10 ×) (New England Biolabs, Inc.), 320 U/mL of Bst 2.0 WarmStart® DNA polymerase, 600 U/mL of WarmStart RTx Reverse Transcriptase (NEB M0380L), 0.1 U/μL of RNase H2 enzyme (catalog: 11-02-12-01, Integrated DNA Technologies), 10 mM of dNTPs (TransGen Biotech), 100 mM of MgSO_4_ (New England Biolabs, Inc.), primers and probe (10 μM). The reaction conditions were optimized for the usage of the cleaved probe. The reaction aimed to detect viral RNA, and the reaction was performed at 1 cycle/min for a total of 60 cycles using a CFX96 Touch real-time PCR detection system (Bio-Rad).

### Specificity of the Cleaved Probe-Based Reverse Transcription Loop-Mediated Isothermal Amplification Method

Under the optimized reaction conditions, the specificity of the CP-RT-LAMP method was tested using the genomic RNA or DNA of PDCoV, PEDV, TGEV, PRRSV, CSFV, SADS-CoV, PCV2, and PRV.

### Detection Limit of the CP-RT-LAMP Method

Standard RNA was serially diluted 10-fold in nuclease-free water (from 2.5 × 10^6^ copies/μL to 2.5 × 10^0^ copies/μL), and the dilutions were used as template for CP-RT-LAMP to test the sensitivity of the established method. A standard curve was constructed on the basis of the results. All experiments were performed three times independently.

### Assembly of the 3D-Printed, Smartphone-Based Cassette for Visualization Detection

A portable and hand-held 3D-printed device was designed for visual inspection of results; it is a cassette with the lower part containing a power supply area and the upper part containing a detection area. The device is powered by a lithium battery and contains eight LED lights. A 495 nm filter is placed directly above the light, and an Eppendorf tube can be placed directly above the filter. An Eppendorf tube containing the reaction mixture was placed in a metal bath for ensuring a constant temperature reaction and then placed in the cassette. When the power is turned on, 495 nm light excites the fluorophores in the reaction mixture and the results can be visualized using a smartphone.

### Clinical Sample Detection

We tested 100 clinical samples including 48 fecal swab specimens and 52 intestinal specimens collected from pig farms. All these specimens were tested using both CP-RT-LAMP and RT-qPCR techniques (19). RT-qPCR technique was performed on an CFX96 Touch real-time PCR detection system (Bio-Rad) with the following conditions: 1 cycle of 45°C for 10 min, 1 cycle of 95°C for 10 min, and 40 cycles of 95°C for 15 s and 60°C for 45 s.

## Results

### Establishment and Optimization of Basic Reaction System

**Figure 1:**
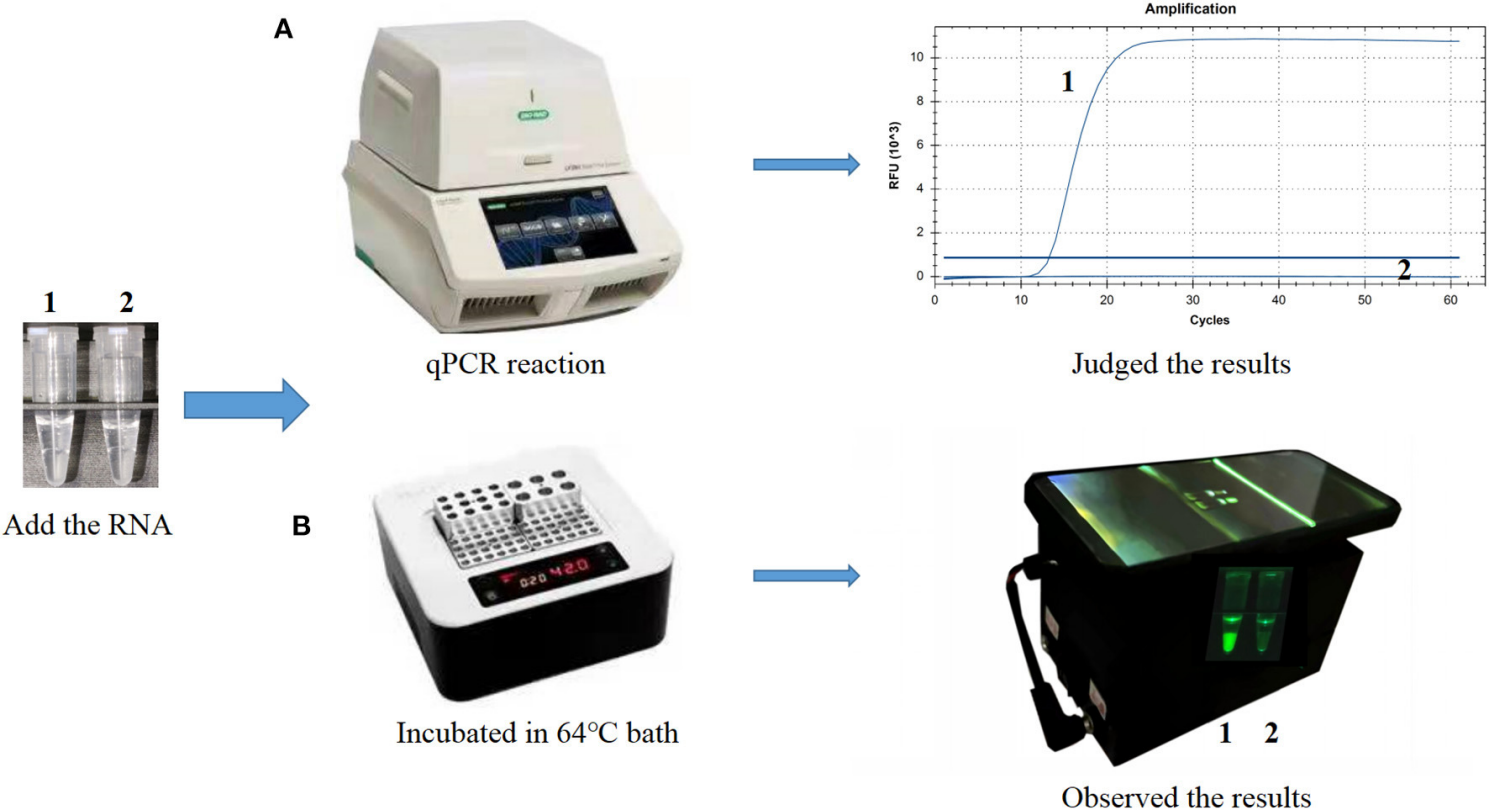
Establishment of the cleaved probe-based reverse transcription loop-mediated isothermal amplification (CP-RT-LAMP) assay for porcine deltacoronavirus. **(A)** CP-RT-LAMP assay using the CFX96 Touch real-time PCR detection system. **(B)** Flowchart of the CP-RT-LAMP method combined with a smartphone-based cassette. Lane or Tube 1, Porcine deltacoronavirus (PDCoV) plasmid. Lane or Tube 2, double-distilled water.

**Figure 2:**
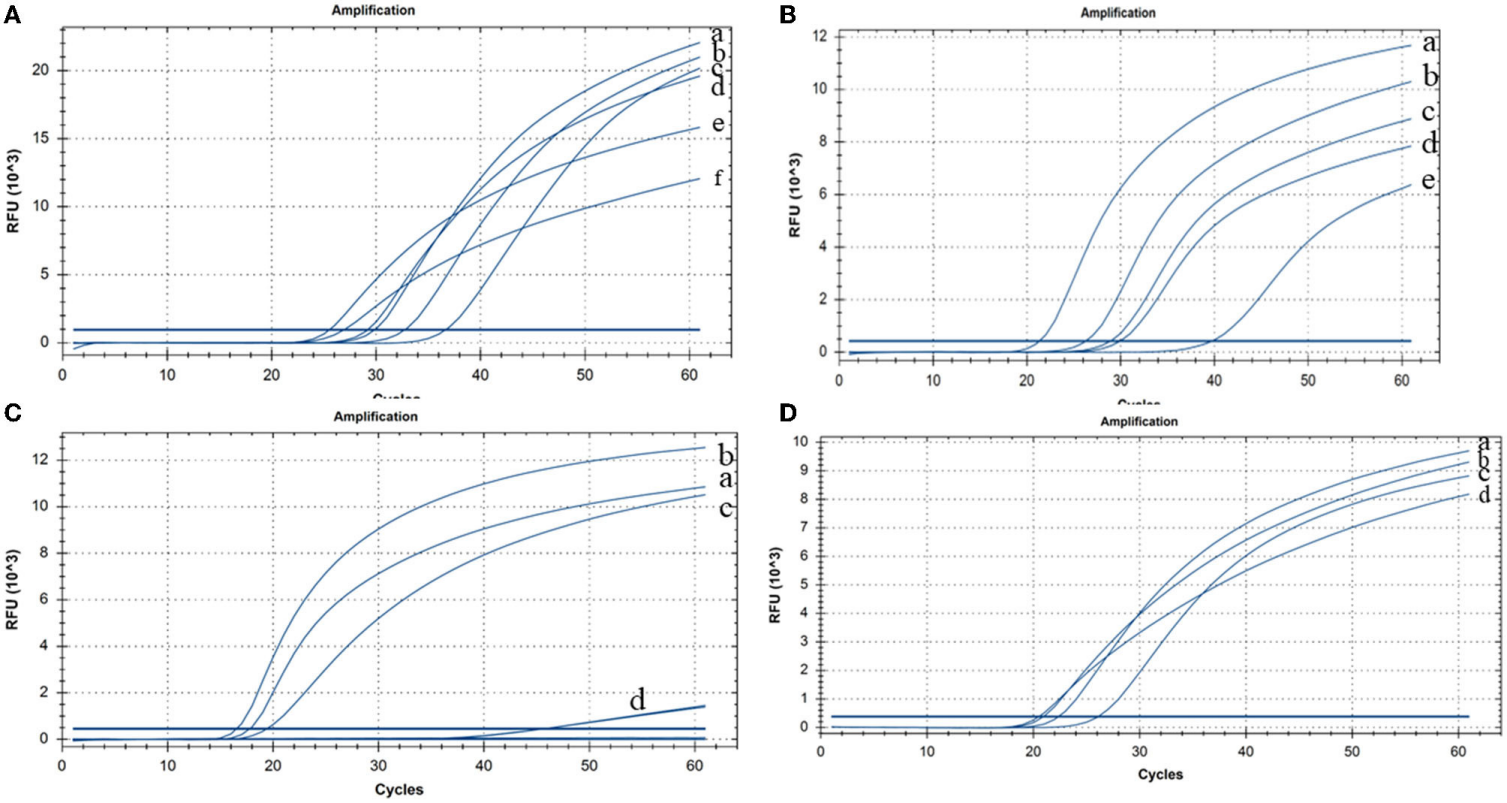
Cleaved probe-based reverse transcription loop-mediated isothermal amplification (CP-RT-LAMP) assay optimization **(A)** Temperature: (a) 63°C, (b) 61°C, (c) 62°C, (d) 60°C, (e) 64°C, and (f) 65°C. **(B)** Concentration of dNTPs: (a) 2.4 mM, (b) 2 mM, (c) 1.6 mM, (d) 1.4 mM, and (e) 1.2 mM. **(C)** Concentration of MgSO_4_: (a) 8 mM, (b) 6 mM, (c) 4 mM, and (d) 2 mM. **(D)** Concentration of the loop primer probe: (a) 0.08 μM, (b) 0.12 μM, (c) 0.04 μM, and (d) 0.16 μM.

The CP-RT-LAMP primers and the cleaved probe were designed on the basis of the conserved sequence of the N gene of PDCoV. When the ribonucleotide of the probe is perfectly complementary to the mutant site, the probe is cleaved by the RNase H2 enzyme, releasing the quencher to provide an amplified signal. Conversely, no signal is generated for a mismatching ribonucleotide. Thus, we successfully validated our principle by establishing a basic reaction system using RNA template of PDCoV. The result not only can be detected by using the portable, hand-held 3D-printed device and smartphone-based cassette for visualization detection, but also detected by using the CFX96 Touch real-time PCR detection system (Figure 1). To optimize the CP-RT-LAMP reaction conditions, amplification was performed at six different temperatures from 60 to 65°C. The amplification curve at 64°C showed the lowest threshold cycle in <40 min, indicating that it was the optimum reaction temperature for CP-RT-LAMP (Figure 2A). At this temperature, the optimal concentration was 2.4 mM for dNTPs (Figure 2B), 6 mM for MgSO_4_ (Figure 2C), and 0.12 μM for the probe (Figure 2D).

### Specificity of the CP-RT-LAMP Method

**Figure 3:**
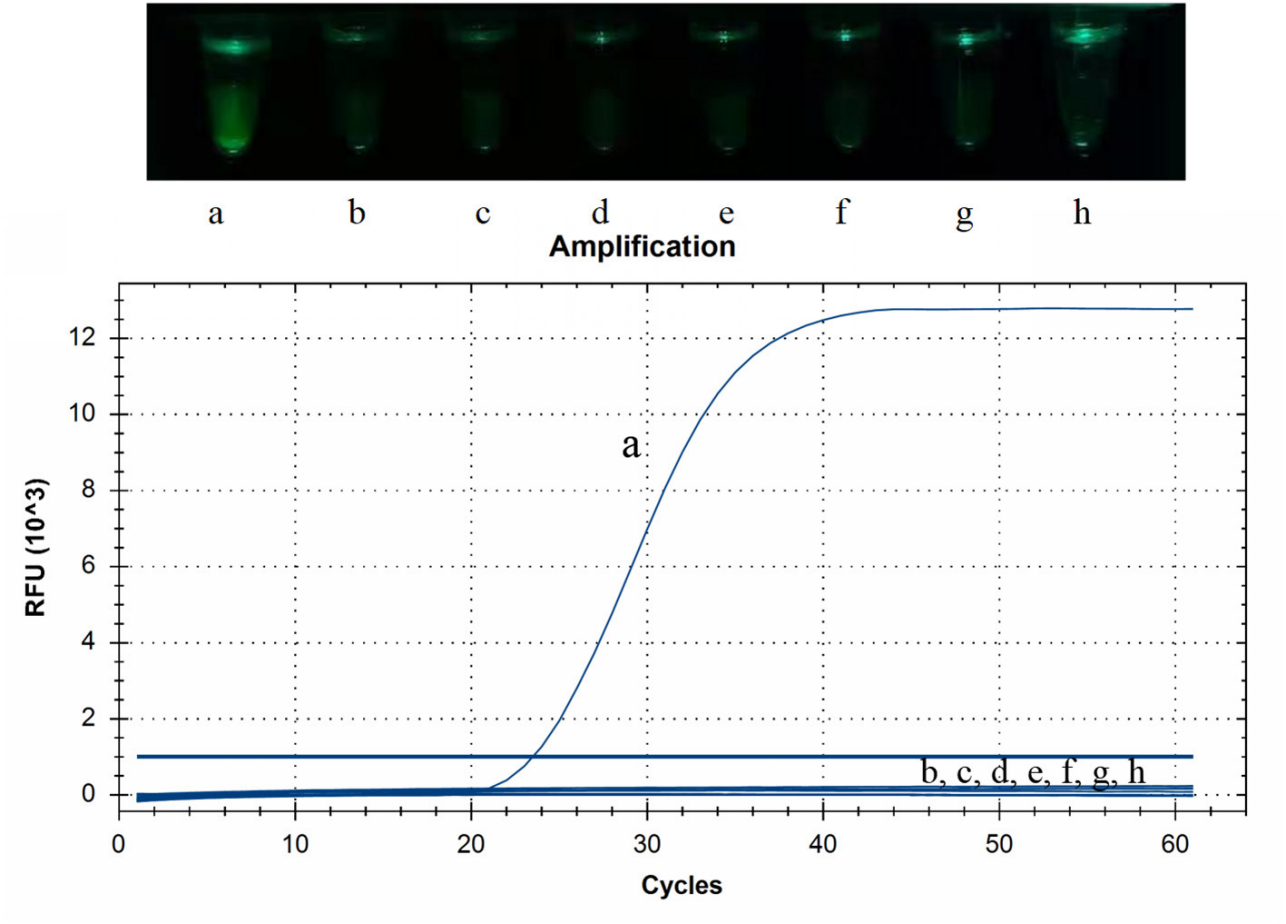
Specificity of cleaved probe-based reverse transcription loop-mediated isothermal amplification (CP-RT-LAMP) assay. Specificity was tested using the genomic RNAs or DNAs of the following viruses: (a) porcine deltacoronavirus, (b) porcine epidemic diarrhea virus, (c) transmissible gastroenteritis virus of swine, (d) porcine reproductive respiratory syndrome virus, (e) classical swine fever virus, (f) swine acute diarrhea syndrome coronavirus, (g) pseudorabies virus and (h) porcine circovirus type 2.

The RNAs or DNAs of PDCoV, PEDV, TGEV, PRRSV, CSFV, SADS-CoV, PCV2, or PRV were tested by CP-RT-LAMP assays. Only PDCoV showed specific amplification, and no amplification products were observed for the other porcine viral pathogens (Figure 3).

### Limit of Detection of the CP-RT-LAMP Method

**Figure 4:**
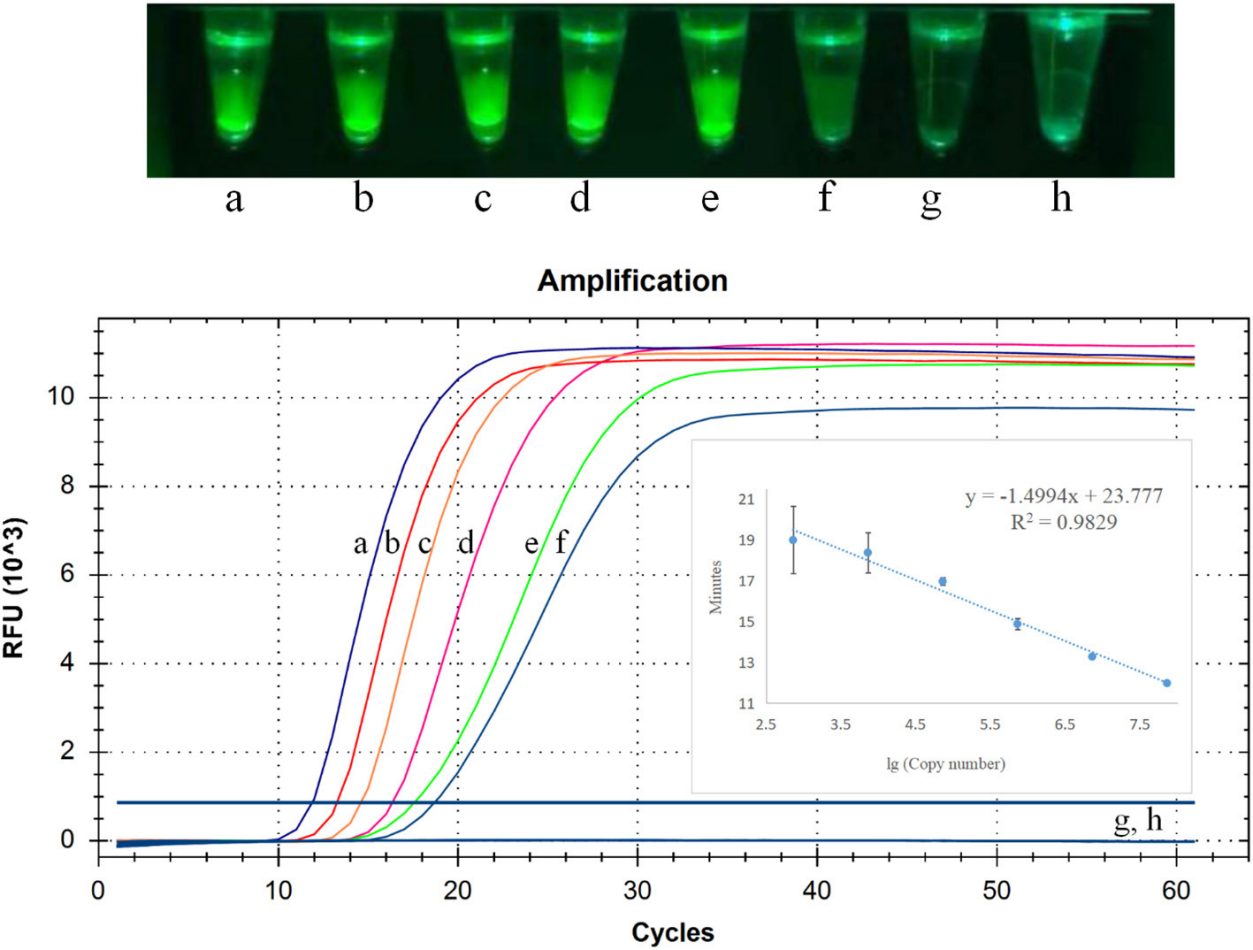
Limit of detection of cleaved probe-based reverse transcription loop-mediated isothermal amplification (CP-RT-LAMP) assay. Serially diluted standard RNA of porcine deltacoronavirus (PDCoV) was used at the following concentrations: (a) 2.5 × 10^6^ copies/μL, (b) 2.5 × 10^5^ copies/μL, (c) 2.5 × 10^4^ copies/μL, (d) 2.5 × 10^3^ copies/μL, (e) 2.5 × 10^2^copies/μL, (f) 2.5 × 10^1^ copies/μL, (g) 2.5 × 10^0^ copies/μL, (h) negative control.

To detect sensitivity of CP-RT-LAMP assay for PDCoV detection, we used 10-fold serial dilutions (2.5 × 10^6^ to 2.5 × 10^0^ copies/μL) of PDCoV standard RNA. As illustrated in Figure 4, the limit of detection of CP-RT-LAMP was 2.5 × 10^1^ copies/μL, which indicates a sensitivity similar to that of RT-qPCR. The standard curve equation was y = −1.4994x + 23.777 (*R*^2^ = 0.9829). When the fluorescence of the reaction mixture under the light of our cassette in the fluorescence product mode was visualized using a smartphone, the limit of detection was 2.5 × 10^1^ copies (Figure 4).

### Application of the CP-RT-LAMP Method in Clinical Samples

To evaluate the application value of this detection method in clinical samples, 100 clinical samples obtained from pigs with diarrhea were collected from different pig farms in China. All samples were examined for the presence of PDCoV using RT-qPCR and CP-RT-LAMP to evaluate the detection efficiency of these two assays. Results indicated that a total of 26 samples were detected as positive by the CP-RT-LAMP method, whereas 17 of these 26 positive samples were detected as positive by RT-qPCR, which indicates that the detection rate of CP-RT-LAMP is comparable to that of RT-qPCR. This finding demonstrates that CP-RT-LAMP can be regarded as a novel diagnostic assay for the detection of PDCoV.

## Discussion

In recent years, PDCoV has spread in many countries, causing huge economic losses to the pig production industry, and it remains a serious challenge (20). The clinical symptoms of PDCoV infections are not easy to distinguish clinically from infections with viruses such as TGEV and PEDV that lead to diarrhea symptoms (21). Therefore, it is necessary to use accurate diagnostic techniques to detect and identify these viruses in a timely manner. In addition, owing to the lack of effective vaccines and treatment (22), the development of a real-time PDCoV detection method is critical to limit the disease spread at an early stage. In the present study, we established a CP-RT-LAMP method that can achieve real-time quantitative detection of PDCoV using fluorescence quantitative PCR. Moreover, this method can accomplish visual detection with a 3D-printed, smartphone-based cassette platform.

At present, molecular diagnostic techniques of PDCoV mainly rely on conventional PCR and real-time qPCR (1, 11, 23). Although these techniques have been widely validated and are useful tools for detecting PDCoV, they are inconvenient because of the expensive instruments and professional operation system required. In the present study, we developed and evaluated the CP-RT-LAMP assay, which showed high specificity and no cross-reactivity with other enteric pathogens. The reaction can be completed in <40 min at 64°C, which greatly shortens the time required for RT-qPCR (19). Rapid detection can save a significant amount of time for epidemic prevention and control. Moreover, the method is highly sensitive, with a detection limit of 25 copies/μL. Its sensitivity was 100 times higher than that of reverse transcription PCR (15, 24), which is comparable to the sensitivity of RT-qPCR in the literature (25). When 100 clinical specimens were tested, CP-RT-LAMP showed a higher detection rate than RT-qPCR. Additionally, the results illustrate that the CP-RT-LAMP method requires less time than real-time qPCR not only for the assay but also the detection process. Previous research demonstrated that a single-tube one-step reverse transcription loop-mediated isothermal amplification (RT-LAMP) assay to detect PDCoV, which analyzed the amplification products by gel electrophoresis or visually detected by adding SYBR Green I dye. But this method can't achieved objective real-time detection and quantitative detection (15). However, our method uses a closed-tube reaction system for detection, thus preventing potential contamination that may be caused by opening the tube after the reaction is complete. Moreover, compared with detection using a fluorescent dye (26), the CP-RT-LAMP method produces fluorescence signals only when the probe is specifically complementary to the target sequence, which reduces the likelihood of false positive results.

Another advantage of CP-RT-LAMP is that it can be performed on a simple, fluorescent quantitative PCR instrument or on-site isothermal instruments, and it facilitates rapid detection of PDCoV in the field. Furthermore, the results can be visualized with a smartphone, which may help in monitoring and preventing outbreaks of PDCoV infection in a timely manner. Moreover, the established the 3D-printed cassette-CP-RT-LAMP detection system's sensitivity is consistent with the results of using fluorescent quantitative instruments, and the limit of detection is 25 copies/μL. Therefore, it is more suitable for rapid on-site monitoring than the conventional methods.

In conclusion, a comprehensive analysis of the CP-RT-LAMP method shows that it is a simple, specific, and sensitive tool for rapid and accurate detection of PDCoV. Moreover, this method is particularly suitable for operation in insufficiently equipped laboratories, making it an ideal method for on-site PDCoV detection in the surveillance and control of epidemics caused by porcine enteric coronaviruses.

## Data Availability Statement

The original contributions presented in the study are included in the article/supplementary material, further inquiries can be directed to the corresponding authors.

## Ethics Statement

This animal study was reviewed and approved by the Ethical and Ethics Commission (Institute of Animal Health, Guangdong Academy of Agricultural Sciences, China). The License Number was SYXK (Yue) 2011–0116. Moreover, sample collecting treatment in this study were performed in accordance with national and local laws and guidelines.

## Author Contributions

HS conceived and designed the experiment. SW wrote the manuscript and carried out the experiment. JH and QL performed the analysis. CZ and ZL prepared materials for the experiments. JZ contributed to sample preparation and collected the data. ML revised the manuscript and funded the project. All authors read and approved the final manuscript.

## Funding

This work was supported by the Key-Area R&D Program of Guangdong Province (2020B0202080004), the Science and Technology Planning Project of Guangzhou (202103000096), Start-up Research Project of Maoming Laboratory (2021TDQD002), the Special Fund for Scientific Innovation Strategy-construction of High Level Academy of Agriculture Science-Prominent Talents (R2020PY-JC001), the Science and Technology Planning Project of Guangdong Province, China (2020A1515010950, 2021A1515011125), and the National Natural Science Foundation of China (31302101).

## Conflict of Interest

The authors declare that the research was conducted in the absence of any commercial or financial relationships that could be construed as a potential conflict of interest.

## Publisher's Note

All claims expressed in this article are solely those of the authors and do not necessarily represent those of their affiliated organizations, or those of the publisher, the editors and the reviewers. Any product that may be evaluated in this article, or claim that may be made by its manufacturer, is not guaranteed or endorsed by the publisher.
